# The role of episodic memory sampling in evaluation

**DOI:** 10.3758/s13423-023-02413-z

**Published:** 2023-11-29

**Authors:** Alice Mason, Gordon D. A. Brown, Geoff Ward, Simon Farrell

**Affiliations:** 1 University of Bath, Bath, UK; 2 University of Warwick, Coventry, UK; 3 University of Essex, Colchester, UK; 4 University of Western Australia, Perth, Australia

**Keywords:** Free recall, Sampling, Retrospective evaluation, Judgment, Episodic memory

## Abstract

Many models of choice assume that people retrieve memories of past experiences and use them to guide evaluation and choice. In this paper, we examine whether samples of recalled past experiences do indeed underpin our evaluations of options. We showed participants sequences of numerical values and asked them to recall as many of those values as possible and also to state how much they would be willing to pay for another draw from the sequence. Using Bayesian mixed effects modeling, we predicted participants’ evaluation of the sequences at the group level from either the average of the values they recalled or the average of the values they saw. Contrary to the predictions of recall-based models, people’s evaluations appear to be sensitive to information beyond what was actually recalled. Moreover, we did not find consistent evidence that memory for specific items is sufficient to predict evaluation of sequences. We discuss the implications for sampling models of memory and decision-making and alternative explanations.

## Introduction

Sampling is a fundamental process in many models of judgment and choice, ranging from Bayesian sampling models (Sanborn & Beierholm, 2016) to episodic reinforcement learning (Bornstein et al., 2017; Gershman & Daws, 2017). Many of these models assume that individual episodes or pieces of information provide the raw materials for evaluation, and that at least some of these samples are drawn from memory (Lindskog et al., 2013). However, few studies have directly tested the extent to which people’s memory for specific events predicts their actual evaluation of an overall experience (e.g., how much they would be willing to pay to go on a holiday or how pleasant they rate a music or film clip to be (Aldrovandi et al., 2015; Kemp et al., 2008; Montgomery & Unnava, 2009)). Here, we report a pre-registered experiment supported by Bayesian mixed effects modeling that directly tests the extent to which people rely on memory-based samples during evaluation.

Early models of memory-based decision-making assumed that, during choice, items are sampled from memory according to their similarity with the current options (Dougherty et al., 1999). More recently, exemplar based models – in which each item is stored in a unique memory trace – have been used to model choice in decisions-from-experience paradigms (Hotaling et al., 2020). In a similar vein, reinforcement learning models have begun to recognize the effects of individual outcomes on choice. In some models of choice (e.g., Erev et al. , 2008; Plonsky et al. , 2015) participants evaluate options using a subset of samples from each option. Such accounts can be classified as episodic reinforcement learning models because individual trials, instead of running averages, are assumed to predict evaluation and choice (Bornstein et al., 2017).

Experimental evidence from the retrospective evaluation literature is consistent with the episodic sampling from memory approach. People performing retrospective evaluation tend to overweight the most intense (“peak”), and the most recent (“end”), events in their overall evaluation of an experience made up of a sequence of events. The peak-end rule has been used to explain evaluations across a range of context, including people’s enjoyment of a meal (Robinson et al., 2011), ratings of film clips (Fredrickson et al., 1993), evaluations of actual holidays (Kemp et al., 2008), ratings of painful medical procedures (Redelmeier & Kahneman, 1996) and people’s financial habits, ranging from purchasing entertainment tickets (Dixon & Verma, 2013) to describing preferences for payment sequences (Langer et al., 2005) and loan repayments (Hoelzl et al., 2011).

To provide a strong test of the episodic sampling from memory approach, we focus here on the direct relationship between individual recalls and evaluations. While previous work has not had the granularity to permit this examination, several studies have linked patterns in judgment to similar patterns in memory, including the better recall of more recent and more extreme events (Aldrovandi et al., 2015; Kemp et al., 2008; Montgomery & Unnava, 2009). Montgomery and Unnava (2009) conducted a series of studies examining how recall of items at the beginning, middle and end of a sequence influenced people’s overall evaluation of the sequence. The sequences were either events describing a vacation or clips of music. To measure evaluation, people were asked how much they would be willing to pay to go on a similar vacation or to listen to a song. The authors introduced a delay between learning and testing, which is known to increase primacy and reduce recency in recall (Bjork & Whitten, 1974; Glanzer & Cunitz, 1966; Postman & Phillips, 1965), and found that this increased participants’ weighting of early presented items in evaluation. Similarly, Aldrovandi et al. (2015) presented people with word lists and found that, when tested after a delay, participants had worse recall of the final items. Their overall evaluation, measured as a pleasantness rating of the word list, was less influenced by a negative item at the end of a list when there was no delay. In both these studies, the focus was on how factors such as valance of the item, task expectancy and item position influenced evaluation.

The present work continues this research program by providing a more fine-grained examination – at the level of multiple individual item recalls, rather than aggregate patterns of recall – of the relationship between memory and evaluation. A key distinction between our experiments and most other retrospective evaluation studies is that we use numbers as the stimuli (but see Langer et al. , 2005; Varey and Kahneman , 1992), allowing us to collect fine-grained measures of memory performance. Furthermore, participants in our experiments were told that the numbers represent values in a fictitious currency. The use of numbers allows us to have precise control over the value of stimuli and consistency in interpretation by participants. Previous research has used words varying in valence, descriptions of people or holiday experiences or subjective measures such as pain (Fredrickson, 2000; Kemp et al., 2008; Lichtenstein & Srull, 1987), which can make it difficult to compare evaluations across participants. Using numerical values also allowed us to measure evaluation in an incentive-compatible fashion.

### The current experiment

Given the importance of sampling in models of memory and choice, but the relative lack of detailed evidence relating to these models, we tested how well people’s memory for individual past experiences predicts their evaluation. We showed participants sequences of numbers that represented fictitious currency values. After viewing each sequence of numbers, participants were asked to recall as many items as possible and to complete an incentivized willingness-to-pay task.

Using model comparison, we examined the ability of three different models to predict evaluation (see Table 1 for a summary of the models). First, a *Recalled items* model was examined, according to which memory for each value is used to predict evaluation. On each trial, the average of the items a participant recalls is used to predict overall evaluation. Second, the *Presented items* model predicts the willingness to pay (WTP) estimate from the average of all presented items – the true value of the sequence. The *Presented items* model makes no assumptions about how the average is calculated, and serves as a comparator for the memory-based model. We examined a value updating model: this *Temporal difference learning* model updates the evaluation at each time step, and effectively produces a recency weighted function across serial positions (Sutton & Barto, 1998; Wulff & Pachur, 2016). Finally, we fitted a variant mixture in which each trial was assumed to be generated from the ‘Recall-based’ model, the ‘Presented items’ model, or the ‘Both’ model, which includes both recalled and presented items as predictors.

**Table 1:** List of models tested

Predictor	*Description*
Presented items	*Average of all values in the sequence*
Recalled items	*The average of all the values recalled for each trial, for each individual*
Temporal difference	*A recency weighted learning rule*
Mixture	*A variant mixture model examining the extent to which individuals use the recalled items, the presented items or both to form their evaluation*

## Method

### Participant recruitment

The experiment was pre-registered (see https://osf.io/se2gg/ for details). Participants were recruited via Prolific Academic to participate in the experiment online. The initial pre-registered sample size was set to 60 based on money considerations and precedent from previous related literature.

To be deemed eligible, participants needed to be aged 18 to 65, have English as their first language (self-reported), be a resident of the UK, USA, Ireland, Australia, New Zealand, or Canada, and have a Prolific Academic approval rating of over 90%. We did not collect data about participants’ education levels. Participants were reimbursed for their time according to the standard rates on Prolific Academic (£5-£6/hour, at the time the experiment was conducted). Participants could earn an additional performance related-bonus between £0 and £2.

### Task design

Participants completed 32 trials. On each trial, participants were presented with a sequence of two-digit numbers. Participants were told that numbers represented amounts in a fictitious currency, Galactic Credits (GC), where 200 GC was equal to £1. Accordingly, each sequence had a currency value that was simply the average of the numbers in that sequence. Each number was presented one at a time for 1500 ms, with an inter-stimulus interval of 1000 ms. After each sequence of numbers, participants completed both the recall task and the evaluation task, with the order counterbalanced across trials.

#### Sequences

The sequences were drawn from a uniform distribution, and the minimum and maximum values varied across each list but were always between 11 and 99. When generating the sequences, the variability within sequences was made large relative to between-sequence variability so that sampling more numbers would be most obviously beneficial to accurately estimate the mean. To achieve this, the mean of each sequence was randomly sampled from the numbers 40 to 70 (a uniform distribution). We then calculated the highest and lowest value for the distribution of each sequence by adding or subtracting 29 from the mean (i.e., each sequence distribution had a range of 58 but different minimum and maximum values). Finally, seven numbers were randomly sampled from this sequence distribution.

#### Free recall task

If a recall cue appeared, participants had 15 s to recall as many items as possible in any order. Participants were instructed to type their responses, pressing enter after each item, into a response box at the center of the screen. The screen was cleared each time the participant pressed enter so that previous responses were not visible. We chose a short recall period of 15 s because Aldrovandi et al. (2015) suggested that when a longer period of 2 min is used participants engage in exhaustive recall, whereas we were interested in the immediately accessible items that would plausibly contribute to evaluation in this setting and in everyday life (Kitayama & Burnstein, 1989). Furthermore, Miller et al. (2012) found that recall is more likely to be self-terminated as output position (and therefore recall time) increases.

#### Evaluation task

An auction procedure was used to obtain participants’ willingness-to-pay (WTP) for another draw from each sequence of numbers, elicited using the bidding procedure developed by Becker et al. (1964). For each trial, and therefore for each bid, participants were given an endowment of 100 Galactic Credits (GCs). Participants state how much they would be willing to pay for a new draw from the sequence (also in GCs) and a selling price was randomly drawn from a uniform distribution of prices (the range of means used in the experiment). If the bid was below the selling price, the participant did not purchase the item and kept only the endowment. If the bid was above the randomly drawn selling price, then the participant automatically used their endowment to buy the sequence at the selling price, and kept the remainder of the endowment. In this latter case, the participants earnings were then the remainder of the endowment, plus the currency value (the average) of the sequence that had been purchased. The optimal strategy – placing a bid equal to the estimated true value of the sequence – was explained to participants. This method is incentive compatible as only the participant’s bid determines whether or not they buy the sequence, and the greatest payoff would be obtained by bidding the believed true value of the sequence (see https://osf.io/92x4w/ for full WTP instructions shown to participants).

### Data analysis

The inferential framework used was Bayesian estimation and model comparison. For the analyses where we are comparing mean recall or WTP across conditions, we used the BayesFactor package (Morey & Rouder, 2022) to estimate Bayes Factors. For these Bayes factor ANOVA analyses, the scale of the effect size for fixed effects ($r$ scale) was set to .707, labeled the “medium” prior in the package. Where we are testing multiple effects, we use the “top” method to estimate Bayes factors, otherwise the default is to compare against a null model that only has a subject-specific intercept. For *t*-tests, the analyses used an uninformative Jeffrey’s prior on the variance, and a standard Cauchy prior of $\sqrt{2}/2$ on the $r$ scale value. For a discussion on priors, see Rouder et al. (2016).

The value of the Bayes factors quantifies the strength of evidence in favor of one model with respect to another given the data obtained. It informs us how much our prior beliefs should shift in response to the data obtained. Although there are no strict cut-offs, we apply the verbal labels suggested by Wagenmakers et al. (2011) to describe our results.

#### Free recall task

To assess participants’ free recall we examined the overall accuracy at each serial position to produce a serial position curve (SPC).

#### Evaluation task

The true value of the sequence was defined as the average of all the values shown. To measure how accurate participants’ judgements were relative to the true mean, we calculated the root mean square error between participants’ bid amounts and the actual sequence values.

#### Predicting evaluation

A primary interest was to compare the item-specific memory model with a baseline model of presented items. Table 1 lists the four models that were examined. The *Presented items* model represents the predictions of an integrator model that averages all values in the sequence, with equal weighting for the values. The *Recalled items* model was a trial-based memory model. The predicted evaluation for a trial was the average of the values recalled for that trial, including both true and false recollections (i.e., intrusions). The *Temporal difference learning* model is a simple value updating model. Each of the stimuli is used to update the estimated value by the difference between the new stimulus and the current estimate. The degree of updating is governed by a learning rate parameter. This temporally weighted average following the last presented number in the sequence is then used as the predictor. Another possibility is that different participants are adopting different strategies to complete the evaluation task. Moreover, although the previous analyses have assumed that either presented items or recalled items determine valuation, it is possible that both do. To examine this further, the *Mixture* model in which each trial was assumed to be generated from the ‘Recall-based’ model, the ‘Presented items’ model, or the ‘Both’ model, which includes both recalled and presented items as predictors. The probability of each model is estimated separately for each person (with a uniform prior across $\theta _{recalled}$, $\theta _{presented}$, and $\theta _{both}$).

We used Bayesian multilevel modeling to predict participants’ WTP estimates using the R package brms (Bürkner, 2017). For the *Temporal difference learning* model we used the R package R2jags (Su & Yajima, 2015). We specified weakly informative priors on the mean of each of the population level effects that were included in the models. For the *Temporal difference learning* the prior for the learning rate parameter was drawn from a uniform distribution (0,1). The ‘Presented items’ model had a single population level parameter which was the average value of the sequence. In the case where there was a single predictor of WTP, e.g., ‘Presented items’, the prior on the slope was set to N(1,0.5). For all models we also included “participant” as a group-level effect on intercept. The prior on the mean intercept was N(0,10). The standard error on the group-level effect (participant) was a half student *t* (3, 0, 10), which is the default setting in the brms package (Bürkner, 2017). For all of the Bayesian analyses using the brms package the warm-up or burn-in period was 1000, with an additional 4000 iterations to estimate the posterior distribution of each parameter; four chains were run with these values. All R-hat values were 1.05 or below indicating good convergence of the chains. For the *Temporal difference learning* model the initial burn-in period was 5000, with an additional 10,000 iterations and four chains.

To compare each of the three models that predict evaluation in our experiments, we selected the model with the highest marginal likelihood. Then, using the Bayes factor function in brms we obtained a Bayes factor for the relative evidence in favor of each of the models relative to the best fitting one. In contrast, the *Mixture* model looks at strategy use within participants and it is not included in these comparisons.^1^

The “Temporal difference learning” required that participants had completed all trials in the experiment. Therefore, as the models were being compared, for all the models only participants who had completed all trials were included in these analyses.

### Results

As detailed in the pre-registration, we excluded five participants as they failed to complete at least 80% of all trials in the experiment. We also had exclusion criteria for the memory and evaluation tasks. Five participants were excluded for poor performance in the memory task (on average less than one item correct per trial). For the evaluation task, nine participants were excluded for not completing at least 80% of the trials. A total of 19 participants were excluded, and 72 were included in the analysis^2^. The age range of the final sample was 18–66 years old and a histogram of participants ages can be seen in the Appendix Fig. 4.

**Fig. 1:**
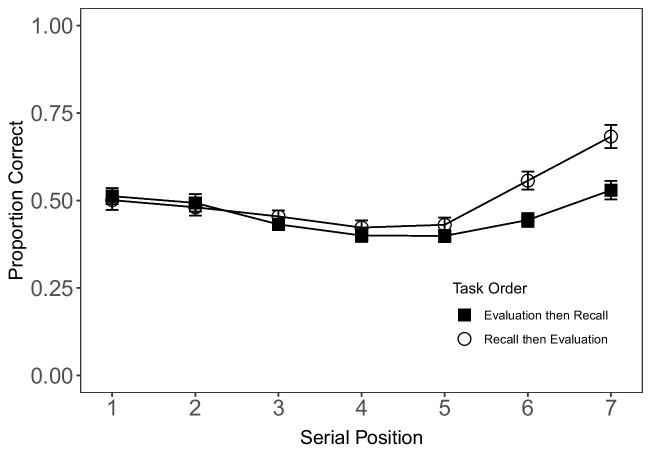
Serial position curves showing the mean proportion of items recalled (+/-SEM) from each task order as a function of serial position. Error bars show standard error of the mean (SEM) within-subject error bars calculated using the method in Morey (2008)

**Fig. 2:**
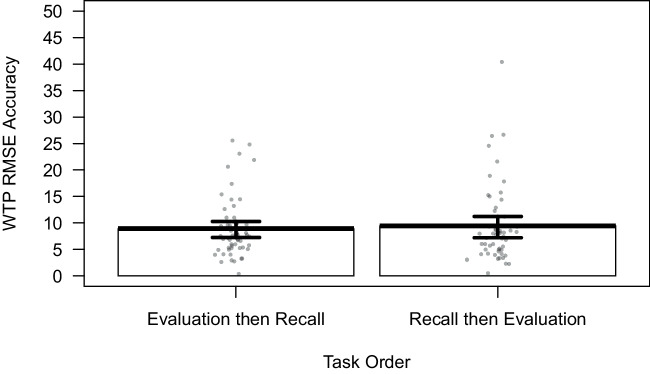
Evaluation task accuracy: Root mean squared error of the willingness-to-pay (WTP) estimate (+/-SEM) in Galactic Credits

#### Memory performance

The recall patterns are as expected for seven-item lists. For both conditions (RE: recall followed by evaluation; ER: evaluation followed by recall) the accuracy serial position functions show a small amount of extended primacy, and recency across the last two serial positions (Fig. 1). Participants’ recall was more accurate in the recall first condition. A Bayesian *t*-test found extreme evidence of an effect of task order on recall accuracy ($BF_{10} =$ $5.15 \times 10^{4}$). To examine the effect of serial position on recall probability we conducted a mixed effects logistic regression predicting recall probability from serial position. This was done separately for each task order. There is extreme evidence of an effect of serial position on accuracy for each of the task order conditions (RE: $BF_{10_{RE}}=$ $6.45 \times 10^{206}$; ER: $BF_{10_{ER}}$ = $1.77 \times 10^{211}$). This second condition is effectively delayed recall and as expected we see reduced recency compared to the RE condition where recall is immediate.

#### Accuracy of evaluation

Participants’ accuracy in the WTP task is shown in Fig. 2. One participant was excluded from all the evaluation analyses as their WTP estimates were outside of the required range (0–100). A Bayes factor *t*-test indicates anecdotal evidence against the effect of task order on WTP estimates ($BF_{order}=$ 0.33).

#### Joint analysis

Because we have memory and evaluation data from each participant for each trial, we can use the ‘Recall-based’ model to test whether memory for a specific set of items predicts evaluation. Thirty-three participants completed all trials, and their data were included in the following analysis. In this scenario, we see extreme evidence in favor of the ‘Presented items’ over all the other models (see Table 2 for details). We pre-registered a secondary analysis to examine whether the memory-evaluation relationship is modulated by task order (see Table 3 for full model fits). We see a small effect of task order in that analysis: When the evaluation task is completed first the ‘Presented items’ model is the best predictor of evaluation; when the recall task is first there is substantial evidence that the ‘Recalled items’ model is a better predictor.

**Table 2:** Bayes factor model comparisons for main analysis

Model name	Bayes factor
Recalled items	$4.29 \times 10^{12}$
Presented items	*
Temporal difference	$1.24 \times 10^{15}$

The first column lists each of the models run in the analysis for the experiments. The model marked with a * has the highest marginal likelihood and the Bayes factors are calculated with respect to that model. The higher the Bayes factor the more evidence there is in favor of the best-fitting model

**Table 3:** Bayes factor model comparisons for task order analyses

Model name	Bayes factor
Evaluation - Recalled items	$7.18 \times 10^{10}$
Evaluation - Presented items	*
Evaluation - Temporal difference	$4.50 \times 10^{1}$
Recall- Recalled items	*
Recall - Presented items	$1.14 \times 10^{1}$
Recall - Temporal difference	$1.72 \times 10^{12}$

One question is how accurate the Bayes factor comparisons are. In other words, what is the likelihood of correctly recovering a model when the true generating model is known? To address this, we report a confusion matrix for the models. We simulated data from each of the models and then fitted each of the models to the simulated data. We noted which model had the lowest marginal likelihood and counted the number of times (out of 50) that each model won. The results are summarized in Table 4. There was perfect recovery for all models.

##### Individual differences

**Fig. 3:**
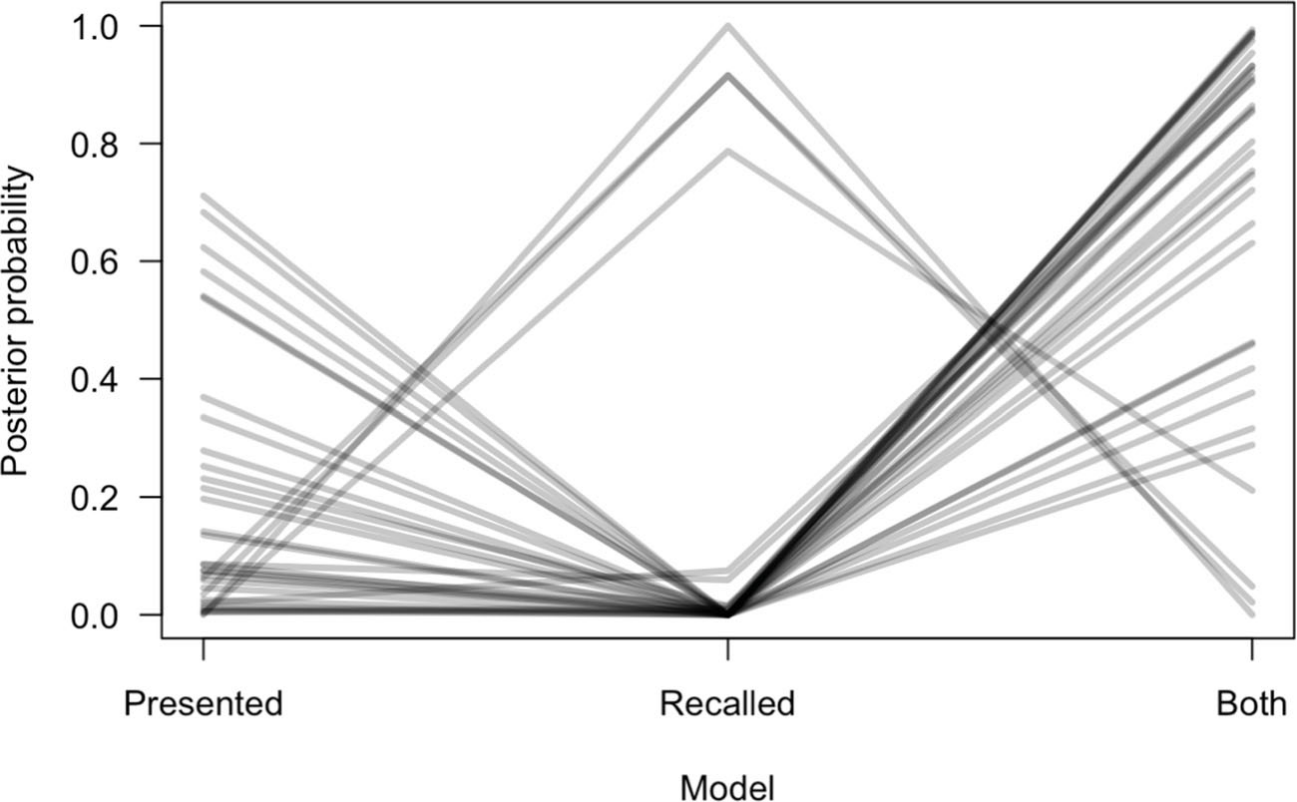
Posterior probability estimates from the variant mixture model which are estimated separately for each participant

An important question is whether different people are using different strategies, or perhaps a mixture of strategies within participants. To address this question, we fit a variant mixture model in which each trial was assumed to be generated from the ‘Recall-based’ model, the ‘Presented items’ model, or the ‘Both’ model, with the probability of each model being estimated separately for each person (with a uniform prior across $\theta _{recalled}$, $\theta _{presented}$, and $\theta _{both}$). Figure 3 plots the posterior probability estimates for each model, and shows that the conclusions at the individual level are broadly compatible with the aggregate model: while a few participants are estimated to primarily rely on a recall-based strategy (*n* = 4), the large majority behave in line with the ‘Presented’ (*n* = 6) or ‘Both’ (*n* = 23) model. The regression estimates (posterior mean and credible interval) in these models were $\beta _{recall}=0.541$ (0.42–0.66) and $\beta _{presented}=0.936$ (0.47–1.50) for the respective individual models, and $\beta _{recall}=0.41$ (0.26–0.57) and $\beta _{presented}=0.54$ (0.33–0.68) for the ‘Both’ model.

**Table 4:** Confusion matrix for model simulations

Generating model	Recalled items	Presented items	Temporal difference
Recalled items	50	0	0
Presented items	0	50	0
Temporal difference	0	0	50

### Discussion

How well do individual samples of past experiences drawn from memory predict evaluations? To answer this question, we collected both memory and evaluation data on each trial. This enabled us to directly assess the influence of memory on evaluation and to test a model that predicted evaluation on the basis of recall on individual trials. The recall model included all recalls (i.e., both true and false recollections). Overall, we did not find evidence that people use individual recalls as the sole basis of their form evaluations, with a relationship only being found when the recall task was conducted immediately before the evaluation task. Instead, at the group-level the unweighted average of the true (i.e., presented) sequence was the best predictor of participants’ evaluations. At the individual level, the majority of participants strategies are best described by the presented model or a model that contains both the presented and recalled items. Our results pose a significant challenge to models that assume evaluations are formed *only* on the basis of distinct episodes drawn directly from episodic memory.

While our results do not establish a strong link between memory and evaluation, the patterns of free recall observed are consistent with existing findings. Few studies have examined patterns of free recall of numbers (Dale & Baddeley, 1966), and the results add to our understanding of the recall of value-based information and its relationship to evaluation. The recall patterns were as expected for seven item lists; we observed one-item primacy and graded recency. Using standard manipulations from the free-recall literature our experiments indicate that recall patterns of numbers follow well-established patterns (Bjork & Whitten, 1974; Farrell, 2012; Grenfell-Essam & Ward, 2015; Postman & Phillips, 1965; Spurgeon et al., 2014; Stefanidi et al., 2018; Ward et al., 2010), though with some quantitative exceptions. We manipulated task order and effectively created an immediate and delayed recall task, and in line with previous work we see evidence of more recency in immediate compared to delayed recall (Bjork & Whitten, 1974). We did observe a flatter serial position function and reduced primacy, which raises the question of whether there is something “special” about the recall of digits as opposed to words. This was examined to some extent by Dale and Baddeley (1966), who found that people have a tendency to recall digits in ascending or descending order. Although our results do not suggest that recall of digits is notably different from recall of words, organizational differences may modulate the expression of recall mechanisms in patterns of recall.

When memory is considered in relation to evaluation, the results challenge prominent models of memory and decision-making that assume the individual samples of past experiences form the basis of evaluation and choice. A unifying feature of these models is that people base their evaluations on discrete samples from memory. These samples could include both correct memories and memory errors. In addition, several samples may be blended together. For example, in instance-based learning individual episodes are retrieved from memory according to their accessibility and are blended together to inform choice (Gonzalez et al., 2003). Similarly, contingent-sampling based models a set of most recent experiences is used to predict upcoming choice (Hochman & Erev, 2013). Notably, the assumption that memory for individual experiences guides evaluation extends beyond psychological models of evaluation and choice (Bornstein et al., 2017; Lieder et al., 2018; Stewart et al., 2006). Reinforcement learning models have introduced an episodic component that keeps an explicit record of past experiences, in contrast to incrementally updating values with experience. These episodes are then weighted according to their similarity with the current decision state (Botvinick et al., 2009; Gershman & Daws, 2017; Lengyel & Dayan, 2007). Our mixture model analysis shows that a small minority of participants did use recall-based strategies and that for the majority of participants the presented items or a combination of both presented and recalled items best predicted their evaluation. Episodic memory sampling models are not sufficient to predict evaluation and need to account for this apparent dual-tasking in people’s strategies.

Our recall-based model provided a superior account of evaluation to any of the other models when recall preceded evaluation. Given that it is not possible to assess memory at the exact moment of evaluation, we decided to counterbalance the task order and to assess memory immediately before and after evaluation. Previous studies have only collected recall data following the evaluation task (Aldrovandi et al., 2015), as conducting recall prior to judgment could artificially boost the relationship by forcing retrieval prior to evaluation (Schwarz & Vaughn, 2002). The accessibility of individual items did change when the recall task was conducted second, and we saw less recency. In cases where samples from memory were generally predictive of recalls this relationship should hold regardless of task order. Our modeling analysis allow us to test whether changes in an items accessibility in memory and the task order affect evaluation. The fact that memory is predictive of evaluation only when the recall task precedes the evaluation task suggests that the recall-evaluation relationship that as observed could be artefactual, and might be explained by recalls acting as a new presentation of items.

Previous work has demonstrated a relationship between patterns of memory recall and evaluation (Aldrovandi et al., 2015; Montgomery & Unnava, 2009). These experiments typically compare features of memory to features of evaluation: for example, when a salient item appears first in a sequence, primacy is observed in both memory and evaluation (Aldrovandi et al., 2015). Similarly, research in impression formation has found that an individual is likely to retrieve recent samples from memory, and this can be related to recency in judgment (Lichtenstein & Srull, 1987). The experimental methods and the modeling used in the present experiment allowed us to provide a more fine-grained account of this relationship by directly assessing the impact of individual recalls from memory on evaluation, in an incentive-compatible paradigm. However, research using a broader range of stimuli (words, film clips, sentences) has indicated that factors including series length, response mode and stimulus complexity can also impact on the strategies people adopt (Hogarth & Einhorn, 1992). Furthermore, people are likely to retrieve, as opposed to construct, underlying preferences in familiar situations (Feldman & Lynch, 1988; Hastie & Park, 1986). This is consistent with our findings that there are individual differences in the strategies adopted by participants. There is a relatively even split among participants who rely on the presented items and participants who additionally use the recalled items. Therefore, although episodic samples from memory alone do not predict evaluation, there can be situations in which memory can support evaluation and the alternative memory representations may play a role (e.g., gist-based: Brainerd et al., 1999; Nosofsky, 1988).

If people are using more than samples from memory to evaluate the sequence, how then are they forming an average value of the sequence? For our analysis, we use a benchmark model, which is the true (unweighted average) value of the sequence. This model was used as a baseline, and was not intended to provide a theoretical account of how people evaluate options. Nonetheless, it is compatible with findings from perception of ensembles, where it is found that people are able to quickly and accurately extract statistical properties from a set of perceptual objects (Whitney et al., 2021). This has been found even when judging numbers presented sequentially, as used here (Brezis et al., 2015; Rosenbaum et al., 2021). Brezis et al. (2015) suggested that people use an analytic, sequential updating strategy for shorter sequences or when not under time pressure, but when information processing is challenged (e.g., fast presentation), they switch to using an automatic and rapid “intuitive” system consistent with ensemble perception. Brezis et al. found evidence of the use of a more analytic strategy using eight-item sequences presented than a faster rate than our sequences, which might seem to rule out the use of an intuitive averaging strategy here. One possibility is that our requirement to also remember the items presented an additional processing burden that pushed our participants into using an intuitive strategy. Considering our findings relating to task order, a compelling question for future research might be whether memory-based evaluation is yet another alternative to analytic and intuitive evaluation (Brezis et al., 2015), the extent to which people adaptively switch between these based on the task environment, and the extent to which estimation is the basis for evaluation (Olschewski et al., 2021).

## Open practices statement

The experiment was pre-registered and the data are available at https://osf.io/se2gg/.

## Figures and Tables

**Fig. 4:**
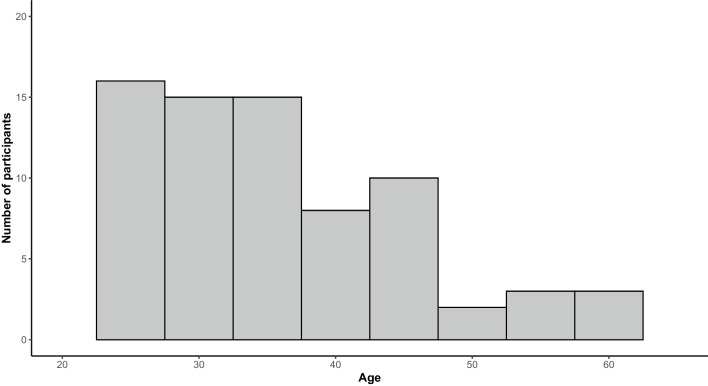
Histogram showing the distribution of participants ages

## Appendix

Figure 4 shows the distribution of ages of the participants who took part in the experiment.

## Footnotes

1 The variant mixture model analysis was not pre-registered and was added in response to a reviewer.

2 The number of participants tested differs from our pre-registered sample size as we report here all the participants who were paid to take part in the experiment. This includes 11 participants who took too long to complete the tasks and timed-out on Prolific Academic
